# Never too late to try something new: attitudes and intention to taste foods from alternative protein sources in a sample of Italian older adults

**DOI:** 10.3389/fnut.2025.1712358

**Published:** 2025-11-18

**Authors:** Maria Elide Vanutelli, Roberta Adorni, Paolo Alberto Leone, Aldo Luperini, Marco D’Addario, Patrizia Steca

**Affiliations:** 1 Department of Psychology, University of Milano-Bicocca, Milan, Italy; 2 Institute of Agricultural Biology and Biotechnology, National Research Council (CNR), Milan, Italy

**Keywords:** automatic attitudes, explicit attitudes, intentions, IAT, plant-based food, cultured meat, insect-based food

## Abstract

**Introduction:**

Nutrition in older adults requires special attention due to protein-energy malnutrition (PEM) risk. Therefore, identifying healthy and sustainable protein sources is crucial, as traditional animal proteins pose challenges to both health and the environment. While most research focuses on younger populations, this study examined the responses of older adults to three alternative protein sources (APS): one plant-based (PBF) and two animal-based sources: cultured meat (CM) and insect-based foods (IBF). We investigated the role of explicit and automatic attitudes in shaping intention to consume (ITC) and the influence of familiarity.

**Methods:**

A between-subjects design was performed: Each participant was randomly assigned to one APS, reported explicit attitudes and ITC, and completed an Implicit Association Test (IAT) to assess automatic attitudes.

**Results:**

Regression analyses showed that ITC varied across APS. For PBF, familiarity was the strongest predictor, followed by explicit attitudes related to taste and automatic attitudes. For CM, ITC was primarily associated with explicit attitudes concerning both taste and safety. For IBF, ICT was mainly related to explicit attitudes concerning taste. ANOVAs comparing the three APSs revealed that IBF was the least favored option. Unexpectedly, although PBF was rated as tastier and safer than CM, it was less preferred in terms of automatic attitudes and ITC.

**Discussion:**

These findings offer new insights into older adults’ openness to APS. Disgust and perceived risk were identified as the primary factors influencing the acceptance of animal-based APS, while familiarity and automatic reactions were key factors in the acceptance of PBF. Importantly, although PBF received positive evaluations at the explicit level, it prompted negative automatic attitudes and low intention to consume, suggesting that older adults may implicitly resist PBF, viewing it as less compatible with their dietary habits compared to CM. This evidence challenges the common belief that PBF is the most accepted category of APS and highlights the need to investigate further the implicit barriers that may prevent the integration of these foods for healthy aging.

## 1 Introduction

### 1.1 Nutrition in the older adults

The average age of the population is steadily increasing. In the last two decades, the average age of the Italian population increased from 42.3 to 46.6 years. The aging of the population is expected to become even more pronounced over the next two decades (1). This phenomenon, while encouraging in terms of life expectancy, raises some critical issues, including health risks, among the older segment of the population. The aging process is accompanied by changes that increase the risk of malnutrition, either in terms of undernutrition or overnutrition of macronutrients and/or micronutrients (2). Protein-energy malnutrition (PEM) is one of the most common forms of malnutrition, often underdiagnosed in the older adults (3–5). PEM is a debilitating condition that results from a decrease in energy intake, particularly from protein sources. It can have serious consequences for health and quality of life (6) and represents a predisposing condition to sarcopenia (7), thus suggesting the need to increase protein intake in older individuals (5).

However, traditional meat-based protein sources have been associated with an increased risk of age-related diseases (8, 9), a decline in physical functioning (10), and frailty (11). Replacing one portion of red meat per day with other protein sources has been associated with a significantly lower risk of frailty (11). In older adults, malnutrition is also linked to difficulties in chewing, particularly with meat compared to other foods (100). From an environmental perspective, the production of meat is associated with environmental issues related to over-exploitation of land and excessive water and carbon footprints (12, 13).

These dual concerns underline the need to explore sustainable protein alternatives that support both health and planetary well-being. Understanding the psychological promoters and barriers toward the consumption of alternative protein sources (APS) represents a promising avenue for fostering virtuous lifestyle habits that can promote healthy aging.

### 1.2 Alternative plant-based protein sources

One possible solution is to switch to predominantly plant-based diets and limit animal-based foods, thereby reducing environmental impact while also improving health outcomes (14). Several studies have been conducted to investigate the effects of a predominantly or totally plant-based diet in the older population, highlighting a positive impact. The most relevant results relate to improved longevity and quality of life (15), reduced risk of frailty (16) and cardiovascular disease (17, 18), an improved body fat composition (19), as well as the prevention of other chronic diseases, such as type 2 diabetes and certain types of cancers (20).

These findings suggest that a diet rich in plant-based sources should be recommended as a dietary strategy to support healthy aging. However, plant-based protein sources have long been considered less nutritionally effective than animal sources due to their incomplete amino acid profile (21, 22). At the same time, it has been demonstrated that a vegan diet can meet nutrient requirements as long as it is well planned in terms of energy needs, variety, and supplementation (3, 20). However, when it comes to an aging population, it is essential to consider whether this is sufficient to address impaired protein metabolism and reduced muscle protein synthesis, indicating a need for high-quality protein. A recent systematic review (23) compared the effects of plant-based protein interventions (ranging from 12 weeks to 1 year) with animal-based protein or non-protein diets on body composition, strength, and physical function in older adults, revealing no significant differences.

Although a diet rich in plant sources is not only possible but advisable for the aging population, some resistance still prevents their consumption. A recent qualitative study (24) aimed at identifying positive and negative beliefs about consuming PBF in a sample of older adults over 65 years of age revealed that health concerns were among the most cited barriers. In particular, they expressed health concerns about the nutritional values of PBF (lack of important minerals, vitamins, or insufficient protein intake), as well as difficulties with digestion (gas and bloating). Then, the complexity of preparation, taste concerns, and lack of satisfaction have been mentioned the most, along with a lack of motivation.

Other alternative sustainable sources of protein include animal-based products, such as cultured meat and insect-based food.

### 1.3 Alternative animal-based protein sources: cultured meat

Cultured meat (CM), also known as cultivated or lab-grown meat, is produced by growing animal muscle cells harvested through a biopsy and then cultivated using a nutrient-rich culture medium that supports cell growth and tissue development (25). Although still not available in most markets due to technical and regulatory barriers, Singapore approved the sale of cultured chicken nuggets in 2020, marking a significant milestone in the commercial application of this technology (26).

CM presents several potential environmental advantages compared to conventional meat. According to early analyses, CM could reduce land usage by up to 99%, water usage by 96%, and energy consumption by up to 45% (27), although subsequent research has shown less promising results (28). CM also provides nutritional and ethical advantages. Although comparative data on the nutritional composition and safety of CM compared to conventional meat for the general population are not yet available (29), recent research considers it potentially healthier due to its lower fat content and the ability to enrich the product with beneficial nutrients during the cultivation process (30). Additionally, it is free from the antibiotics increasingly found in farmed meat (31). Finally, it eliminates the need to raise and slaughter animals (32).

Despite its benefits, CM faces several challenges related to public acceptance. In general, consumer hesitancy is driven by perceptions of unnaturalness, disgust, and ethical ambiguity (33). A study conducted on an Italian sample (25) found that young, educated meat-eaters who were somewhat familiar with the concept were more receptive than older participants. Indeed, over 60% of participants aged 65 and above declared they were not even willing to try CM. A recent study (34) demonstrated that CM may find acceptance even among older consumers if communication strategies are appropriately designed. The authors found that priming participants with emotions related to regret increased their willingness to try CM, especially among older individuals. They inferred that older adults might be more attuned to the loss associated with inaction, making them more susceptible to loss aversion.

### 1.4 Alternative animal-based protein sources: insect-based food

In recent years, entomophagy—the practice of eating insects—has gained scientific and public attention for its promising environmental, nutritional, and socio-economic potential (35, 36). The benefits of insect farming for the environment include a reduced impact on greenhouse gas emissions, as well as lower land and water use (37). Their ability to thrive on organic waste further enhances their ecological role, contributing to nutrient cycling and waste reduction (38). Regarding the nutritional value of insects, high-quality proteins, essential amino acids, vitamins, and minerals are most commonly cited (39). For some species, the nutritional profile is comparable to conventional meat (40). Additionally, they are a good source of fiber (41), which supports gut microbiota balance and may possess antioxidant properties (42). Insect-based foods (IBF) are also advantageous due to their low caloric content, which may contribute to the prevention or management of chronic conditions such as cardiovascular diseases and obesity (43).

Despite these advantages, the approach to IBF in Western countries remains limited. Previous studies have emphasized the influence of emotional and affective factors in shaping negative attitudes, particularly disgust (36, 44, 45) and risk perception (46, 47). The notion of consuming insects is often associated with feelings of uncertainty and concerns about food hygiene, including potential adverse outcomes such as disease transmission (48). Previous studies have also emphasized the role of gender in influencing the acceptance of alternative protein sources, although the results are conflicting. Most studies indicate that men tend to show greater acceptance compared to women of CM (3, 49), IBF (3, 35, 42), and PBF (24). However, some studies have not identified a significant effect of gender on the acceptance of CM (3, 30) and PBF (3). This skepticism is even more pronounced in the older segments of the population, who are more reluctant to try new foods (33, 50, 51). In their research paper evocative titled “Elderly Resistance vs. Youthful Acceptance,” Castro-Alija et al. (35) revealed that older participants showed greater resistance to incorporating insects into their diets, while showing openness in survival scenarios.

### 1.5 The present study

The present study is situated within this theoretical framework, aiming to investigate from a psychological perspective the factors most closely associated with openness toward APS, with a particular interest in the intention to consume (ITC). We focused on the role of attitudes, given their well-established connection with behavioral intentions (52, 53). According to the Theory of Planned Behavior (52), among other factors, favorable attitudes substantially increase the likelihood of forming a corresponding intention, which in turn predicts the actual enactment of the behavior. In the context of food consumption, positive attitudes toward specific products have consistently been shown to enhance consumers’ willingness and intention to incorporate them into their diets, including APS (30, 54, 55).

The study addresses important gaps in the literature on alternative protein sources (APS) by focusing specifically on the aging population. This group is often underrepresented in this field, despite being particularly vulnerable to protein-energy malnutrition. Previous research has predominantly introduced this group as part of mixed-age samples, often constituting only a minor segment (56). Instead, younger adults have been more frequently targeted as future consumers and recipients of communication interventions (57). However, the older adults could also benefit from appropriate tools and tailored information to introduce safe foods into their diets that are beneficial for metabolism and maintaining muscle mass.

A second novelty lies in its methodological approach. While prior research on psychological determinants of food choice in older populations has mainly relied on self-reported, explicit measures (24, 25, 34), we supplemented them with implicit measures of automatic attitudes. Specifically, we employed the Implicit Association Test (IAT) (58), which has proven effective in capturing automatic processes in food-related contexts, particularly when controversial products are involved, such as plant-based alternatives (59), cultured meat (60), and edible insects (36).

Finally, the study incorporates the role of previous experience with PBF, as familiarity has been linked to improved acceptance (61) and the intention to try novel or unfamiliar foods (62). Yet, the role of previous experience with PBF in modulating attitudes still needs to be clarified in older omnivores.

Based on these premises, the study addresses two main research questions:

*RQ1*: Which factors are most strongly associated with the intention to consume each APS (PBF, CM, IBF)? How does previous experience with PBF contribute?

*RQ2*: How do the three APSs compare in terms of psychological determinants of intention, highlighting their unique profiles of acceptance among older adults?

To address these research questions, we developed an online survey in which a sample of Italian older adults over the age of 65 was randomly assigned to evaluate one of the three APSs. Participants were asked to report their attitudes and ITC the assigned APS, and to complete the IAT.

Based on existing literature, we expected more negative attitudes toward IBF, which typically involve strong emotional reactions (i.e., disgust), compared to other sources of alternative proteins (56, 63–65). We also expected that previous experience with PBF could lead to even more positive attitudes and favorable intentions (61, 62). Moreover, we expected a distinct profile for CM and IBF, with fear being more prevalent for the former (30, 49, 66), and disgust being more prevalent for the latter.

Finally, considering the specific novelties of the present study, we predicted that in our older adult sample, unlike previous findings from younger populations, CM could be perceived as more appealing than PBF. Indeed, for this age group, products such as tofu and seitan—the focus of our survey questions—may have been regarded as relatively novel foods, given their recent introduction to the market. By contrast, although cultured meat may elicit skepticism and fear, it could appear more consistent with a dietary style that remains closely tied to tradition (67).

## 2 Methods

### 2.1 Participants

This study is part of a broader research project aimed at investigating the socio-demographic and psychological factors that influence healthy and sustainable food choices. Participants were recruited through the Bilendi online platform,^1^ a panel provider offering innovative solutions for the collection and management of both quantitative and qualitative research data in Europe and the USA. Financial incentives were provided to encourage participation. Participants were selected based on the following criteria: (1) adherence to an omnivore or flexitarian diet; (2) being between the ages of 65 and 75, with gender matched among participants. Participants assigned to either the CM or IBF versions were selected based on an additional criterion: they must have never tasted the target food. The sample comprised 311 individuals (see Table 1), consisting of 155 women (49.8%) and 156 men (50.2%), with a mean age of 69.90 (SD = 2.77). Over half of the participants (75.9%) held a high school diploma and were retired (77.2%).

**Table 1:** Sociodemographic characteristics of the sample (*n* = 311).

Sociodemographic variables	Plant-based alternatives (*n* = 107)	Cultured Meat (*n* = 102)	Insect-based food (*n* = 102)
Age, mean (SD)	69.8 (2.83)	69.8 (2.87)	69.9 (2.62)
Gender, *n* (%)			
Male	53 (49.5%)	51 (50.0%)	52 (51.0%)
Female	54 (50.5%)	51 (50.0%)	50 (49.0%)
Employment status, *n* (%)			
Working	25 (23.4%)	27 (26.5%)	19 (18.6%)
Retired	82 (76.6%)	75 (73.5%)	83 (81.4%)
Educational level, *n* (%)			
High school or less	79 (73.8%)	80 (78.4%)	77 (75.5%)
Higher than high school	28 (26.2%)	22 (21.6%)	25 (24.5%)

The adequacy of the sample size was determined through power analysis (68) using G*Power Version 3.1.9.7 (69). We calculated the sample size required to perform a linear multiple regression model with the following parameters: f2 = 0.15 (medium effect size), *α* = 0.05, power = 0.80; number of predictors = 5. The calculated sample size required was 92 individuals. Moreover, we calculated the sample size required to perform a one-way ANOVA based on the following parameters: *f* = 0.25 (medium effect size), α = 0.05, power = 0.80; number of groups = 3. The calculated sample size required was 159 individuals.

The research was conducted in accordance with the Declaration of Helsinki and received approval from the ethical committee of the University of Milano-Bicocca (Protocol no. 0180063). Each participant provided written informed consent.

### 2.2 Materials and procedure

Three parallel forms of an online questionnaire were developed, corresponding to distinct food categories. Participants were randomly assigned to one of the food categories, resulting in a between-subjects design. The questionnaires were constructed using the Qualtrics platform and were made accessible through both mobile devices and computers in November 2024. The IAT was implemented through an open-source web app designed for Qualtrics (70, 71).

Participants were first asked to give their informed consent. This section included information about the study’s aims, procedures, duration, and the researchers’ contact details. Next, participants answered questions regarding their socio-demographics, including age, gender, education, and employment status. They were prompted to declare their dietary preferences, choosing among five multiple-choice options the one that best represented their usual eating habits. The options included: “I regularly consume animal proteins, from red meat, white meat, fish, eggs or dairy products,” representing an omnivorous dietary pattern; “I follow a mainly vegetarian diet, without giving up sporadically consuming proteins of animal origin” which indicated a flexitarian dietary pattern; “I eat fish, but I do not eat meat” which indicated a pescatarian dietary pattern; “I do not eat meat nor fish, but I do eat eggs and dairy,” which corresponded to a vegetarian diet; “I do not eat meat and fish, nor do I consume animal source products” which represented veganism [taken and modified from De Backer & Hudders (72)]. As mentioned above, participants who identified as pescatarians, vegetarians, or vegan were excluded.

Participants were also asked about their experiences with their target food category. In the case of CM and IBF, this question was used to screen and exclude participants with previous experience. In the case of PBF, the question was used to categorize into two distinct groups during the data analysis: those who had tried PBF and those who had not.

The final part of the online questionnaire assessed three outcome measures:

- Intention to introduce the food category in the diet
- Explicit attitudes toward the food category
- Automatic attitudes toward the food category

Four *ad-hoc* items were used to gauge participants’ willingness to incorporate four different products into their diet. IBF options included grasshopper flour, cricket burger, larvae cookies, and insect crackers. PBF included vegan cold cuts, tofu burger, seitan ragù, and soy sausage. An example item is: “Do you think you might introduce -product- into your diet in the future?” The response options ranged from 1: “extremely unlikely” to 10: “extremely likely.” In the case of CM, only one item was used to represent the general category. The responses to the four examples within each food category of IBF and PBF were averaged (Cronbach’s alpha was 0.97 for IBF and 0.94 for PBF).

Explicit attitudes toward each product were measured by asking participants to think about it and evaluate it on a 7-point Likert scale using two pairs of adjectives within a semantic differential scale adapted from Maggino and Mola (73). The adjectives used were: “Risky” vs. “Safe” and “Disgusting” vs. “Tasty.” An example item was “What adjectives do you think are most suitable to describe -product-?” A higher score indicated a more positive attitude toward the food category. A mean score was calculated for each couple of adjectives. All scores showed good internal consistency (Cronbach’s alpha ranged from 0.88 to 0.95). In the case of CM, only one item was used to represent the general category.

**Figure 1:**
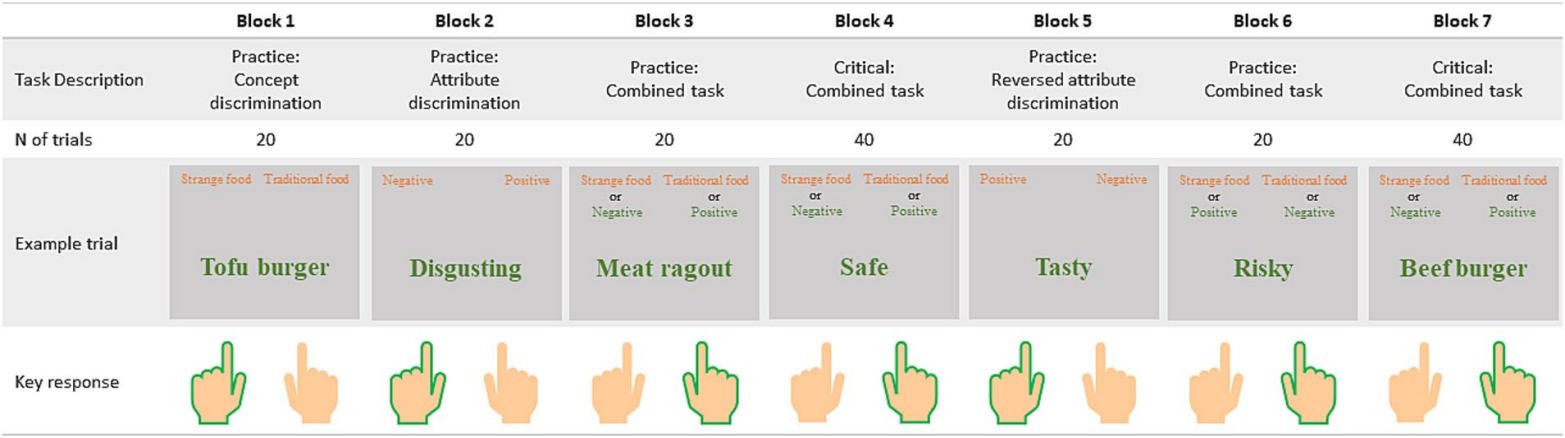
Example of the IAT structure. The protocol includes four counterbalanced conditions to mitigate potential order effects related to category labels. In summary, if a participant follows the pattern outlined in the table, subsequent participants will respond to the concept “plant-based food” or the negative attributes by pressing the right-key button.

To identify automatic associations between each alternative food category, traditional food, and positive or negative attributes, participants completed an adapted version of the Implicit Association Test (IAT) (74, 75). In this task, participants were prompted to associate eight adjectives with either positive or negative valence, with eight words representing alternative or traditional food. Figure 1 illustrates an example of the task structure. Stimuli are listed in Table 2.

**Table 2:** List of the items used to assess automatic attitudes.

Food category	APS	Traditional foods	Adjectives
Plant-based alternatives	Vegan cold cuts Tofu burger Seitan ragù Soy sausage	Pork slices Beef burger Meat ragù Chicken sausage	Bad/Good Risky/Safe Harmful/Healthy Disgusting/Tasty
Cultured meat	Cultured meat (CM) CM roast beef CM filet CM Burger	Raised meat (RM) RM roast beef RM filet RM burger	Bad/Good Risky/Safe Harmful/Healthy Disgusting/Tasty
Insect-based food	Grasshopper flour Cricket burger Larvae cookies Insect crackers	Wheat flour Veal burger Rye cookies Cereal crackers	Bad/Good Risky/Safe Harmful/Healthy Disgusting/Tasty

The underlying assumption was that individuals harboring numerous biases against the alternative food category would find it easier (i.e., exhibit lower response times) to associate it with negative attributes than with positive attributes. Compared to other studies using a more standard version of the task, we created a more focused task that included both traditional and alternative foods to ensure greater ecological value. The strength of the automatic association between the food categories and the positive or negative attributes was quantified by the D-index, which is a score derived from the standardized mean difference between target-attribute pairings that are “inconsistent with the hypothesis” and pairings that are “consistent with the hypothesis” (75). The D-index value typically ranges from −1 to +1. A higher D-index (more positive) indicated a stronger association between pairings consistent with the hypothesis (i.e., the association between the traditional food category and positive attributes). Conversely, a negative D-index suggested a stronger association between pairings inconsistent with the hypothesis (i.e., the association between the APS and positive attributes). A D-index equal to zero indicated the absence of a significant preference for either food category. Errors were managed by requesting participants to correct their responses. Response times and errors were treated according to the guidelines for the improved scoring algorithm (75), and they were automatically calculated through the open-source web app used to implement the task (70, 71).

### 2.3 Data analysis

Analyses were performed using Jamovi (Version 2.3.28, The Jamovi project, 2022, retrieved from https://www.jamovi.org) and IBM SPSS Statistics, version 29 (SPSS, Chicago, IL, United States).

Descriptive statistics were calculated on the sample’s sociodemographic characteristics and the outcome variables. Mean and standard deviation (SD) were reported for continuous variables, and percentages were reported for categorical variables. The normality of the data was tested by calculating the skewness and kurtosis indices; the recommended ranges of ±2 and ±7 were considered for normality, respectively (76). Cronbach’s alpha (77) was calculated to estimate the internal consistency of the synthetic indexes representing the intention to consume the target foods and the explicit attitudes toward them.

#### 2.3.1 RQ1—predictors of the intention to consume APS

Three multiple linear regression models were performed. The dependent variable was the intention to consume each target food (PBF, CM, or IBF). The categorical independent variables included gender (two levels: woman, man) and, for PBF only, tasting experience (two levels: unfamiliar, familiar). Explicit and automatic attitudes served as covariate independent variables. Adjusted R-squared and F-test values were calculated for the explained variance and model fit, respectively.

#### 2.3.2 RQ2—APS comparison of attitudes and intention to consume

Four one-way ANOVAs were conducted using intention to consume, explicit attitudes (“Disgusting” vs. “Tasty,” “Risky” vs. “Safe”), and automatic attitudes as dependent variables. The independent variable in each analysis was the target food (three levels: PBF, CM, IBF). To create three homogeneous groups regarding the familiarity variable, only participants who reported never having tasted PBF were included in the analyses. Before conducting the analyses, the normal distribution of the variables was confirmed through assessments of skewness and kurtosis, and the homogeneity of variances was evaluated using Levene’s test. Based on the results of the assumption checks, ANOVAs were performed using Welch’s Test (for normal distribution and unequal variances) or Fisher’s Test (for normal distribution and equal variances). Post-hoc tests were performed using the Games-Howell Test (unequal variances) or the Tukey Test (equal variances).

All statistical tests were two-tailed, and a *p* ≤ 0.05 was considered statistically significant.

## 3 Results

All the outcome variables were normally distributed (Table 3).

**Table 3:** Descriptive statistics of the outcome variables.

Statistical parameter	APS	Intention to consume	Explicit attitude disgusting/tasty	Explicit attitude Risy/Safe	Automatic attitude (D-index)
Mean	*PBF*	3.94	3.87	4.57	0.82
	*CM*	3.57	3.23	3.24	0.72
	*IBF*	2.66	2.41	2.99	1.10
SD	*PBF*	2.45	1.36	1.63	0.65
	*CM*	3.02	1.87	2.08	0.61
	*IBF*	2.27	1.57	1.85	0.57
Skewness	*PBF*	0.57	−0.21	−0.27	−0.18
	*CM*	0.85	0.26	0.50	−0.21
	*IBF*	1.39	0.73	0.55	−0.24
SE skewness	*PBF*	0.23	0.23	0.23	0.24
	*CM*	0.24	0.24	0.24	0.24
	*IBF*	0.24	0.24	0.24	0.24
Kurtosis	*PBF*	−0.63	0.13	−0.60	1.52
	*CM*	−0.74	−1.03	−1.07	0.02
	*IBF*	0.94	−0.48	−0.80	−0.22
SE kurtosis	*PBF*	0.46	0.46	0.46	0.46
	*CM*	0.47	0.47	0.47	0.48
	*IBF*	0.47	0.47	0.47	0.48

Descriptive statistics revealed that participants generally did not intend to consume APS, particularly IBF. The mean score for IBF was 2.66 (SD = 2.27), CM scored 3.57 (SD = 3.02), and PBF scored 3.94 (SD = 2.45) on a scale from 1 to 10. Overall, participants exhibited a negative attitude, showing a particularly unfavorable response to IBF when assessed on a scale from “Disgusting” to “Tasty,” with a mean score of 2.41 (SD = 1.57) on a range from 1 to 7. Eight participants did not complete the IAT and were subsequently excluded from the analyses. The mean D-Index was 1.10 (SD = 0.57) when the focus was on IBF, 0.72 (SD = 0.61) when the focus was on CM, and 0.82 (SD = 0.65) when the focus was on PBF. As detailed in the procedure section, a positive D-Index indicates a stronger association between positive attributes and traditional foods, while a negative D-Index suggests a stronger association between positive attributes and alternative protein sources. Thus, the results indicated that participants showed a much more favorable automatic attitude toward traditional food than alternative protein sources, particularly when the focus was on IBF.

### 3.1 RQ1—predictors of the intention to consume APS

The multiple linear regression model focusing on the intention to introduce PBF into one’s diet explained 40.8% of the variance and estimated a large effect size (f^2^ = 0.69). A significant regression equation was found [*F*(5, 100) = 15.5; *p* < 0.001]. The results indicated a significant effect of tasting experience (*p* < 0.001), suggesting that familiar participants (Mean = 4.76, SE = 0.27) were more likely to express an intention to incorporate PBF into their diet compared to unfamiliar participants (Mean = 3.08, SE = 0.27). Furthermore, respondents with more favorable explicit attitudes toward the tastiness of PBF (standardized *β* = 0.36, *p* < 0.005) were more likely to incorporate them into their diet. Finally, respondents with more favorable automatic attitudes toward PBF (standardized *β* = −0.16, *p* < 0.05) were more likely to introduce them into their diet (Table 4).

**Table 4:** Multiple linear impacts of gender, tasting experience, explicit and automatic attitudes on the intention to introduce plant-based alternatives in one’s diet.

Predictor	*t*	*p*-value	β	95% CI - Lower limit	95% CI - Upper limit
Intercept	0.502	0.617			
Gender	1.652	0.102	0.2511	−0.0505	0.55269
**Familiarity**	**4.343**	**< 0.001**	**0.6806**	**0.3697**	**0.99146**
**Explicit attitude: Disgusting/Tasty**	**3.208**	**0.002**	**0.3577**	**0.1365**	**0.57895**
Explicit attitude: Risky/Safe	0.523	0.602	0.0584	−0.1633	0.28011
**Automatic attitude (D-index)**	**−2.067**	**0.041**	**−0.1599**	**−0.3134**	**−0.00639**

Significant effects are highlighted in bold.

The multiple linear regression model focusing on the intention to introduce CM into one’s diet explained 70.2% of the variance and estimated a large effect size (f^2^ = 2.36). A significant regression equation was found [*F*(4, 94) = 58.7; *p* < 0.001]. The results indicated a simultaneous significant impact of the explicit attitudes. Respondents with more favorable explicit attitudes toward the tastiness (standardized *β* = 0.46, *p* < 0.001) and safety (standardized *β* = 0.38, *p* < 0.001) of CM were more inclined to introduce them into their diet (Table 5).

**Table 5:** Multiple linear impacts of gender, explicit and automatic attitudes on the intention to introduce cultured meat in one’s diet.

Predictor	*t*	*p*-value	β	95% CI - Lower limit	95% CI - Upper limit
Intercept	−0.140	0.889			
Gender	−1.823	0.071	−0.2017	−0.421	0.0180
**Explicit attitude: Disgusting/Tasty**	**4.411**	**< 0.001**	**0.4621**	**0.254**	**0.6701**
**Explicit attitude: Risky/Safe**	**3.675**	**< 0.001**	**0.3841**	**0.177**	**0.5917**
Automatic attitude (D-index)	−1.466	0.146	−0.0823	−0.194	0.0292

Significant effects are highlighted in bold.

The multiple linear regression model focusing on the intention to introduce IBF into one’s diet explained 56.6% of the variance and estimated a large effect size (f^2^ = 1.30). A significant regression equation was found [*F*(4, 93) = 32.6; *p* < 0.001]. The results suggested that respondents with more favorable explicit attitudes toward the tastiness (standardized *β* = 0.57, *p* < 0.001) of IBF were more inclined to introduce it into their diet (Table 6).

**Table 6:** Multiple linear impacts of gender, explicit and automatic attitudes on the intention to introduce insect-based food in one’s diet.

Predictor	*t*	*p*-value	β	95% CI - Lower limit	95% CI - Upper limit
Intercept	0.0319	0.975			
Gender	0.1033	0.918	0.0139	−0.2529	0.2807
**Explicit attitude: Disgusting/Tasty**	**46.155**	**< 0.001**	**0.5657**	**0.3223**	**0.8091**
Explicit attitude: Risky/Safe	17.728	0.080	0.2188	−0.0263	0.4638
Automatic attitude (D-index)	−0.5671	0.572	−0.0385	−0.1732	0.0963

Significant effects are highlighted in bold.

### 3.2 RQ2—APS comparison of attitudes and intention to consume

The first ANOVA examined the intention to consume APS as the dependent variable. In this and the following ANOVAs, the food category (three levels: PBF, CM, and IBF) was the independent variable. Assumption checks indicated that the variances across groups were not homogeneous [Levene’s test (2, 250) = 18.73; *p* < 0.001]. The results revealed a significant effect of food category [Welch’s *F*(2, 152) = 3.79; *p* < 0.05; *f* = 0.49]. The intention to consume IBF (Mean = 2.57; SD = 2.14) was significantly lower (*p* < 0.05) than the intention to consume CM (Mean = 3.59; SD = 3.03). The intention to consume PBF was in the middle (Mean = 2.82; SD = 1.81).

The second ANOVA examined explicit attitudes on the Disgusting / Tasty scale as the dependent variable. Assumption checks indicated that the variances across groups were not homogeneous [Levene’s test (2,250) = 9.41; *p* < 0.001]. The results revealed a significant effect of food category [Welch’s *F*(2, 149) = 14.66; *p* < 0.001; *f* = 0.50]. IBF (Mean = 2.34; SD = 1.50) was rated significantly (*p* < 0.001) more disgusting than CM (Mean = 3.22; SD = 1.87) and PBF (Mean = 3.56; SD = 1.33).

The third ANOVA examined explicit attitudes on the Risky / Safe scale as the dependent variable. Assumption checks indicated that the variances across groups were not homogeneous [Levene’s test (2,250) = 6.85; *p* < 0.001]. The results revealed a significant effect of food category [Welch’s *F*(2, 148) = 9.83; *p* < 0.001; *f* = 0.46]. IBF (Mean = 2.90; SD = 1.81) and CM (Mean = 3.28; SD = 2.09) were rated significantly (*p*-values was < 0.001 and < 0.005 respectively) less safe than PBF (Mean = 4.13; SD = 1.57).

**Figure 2:**
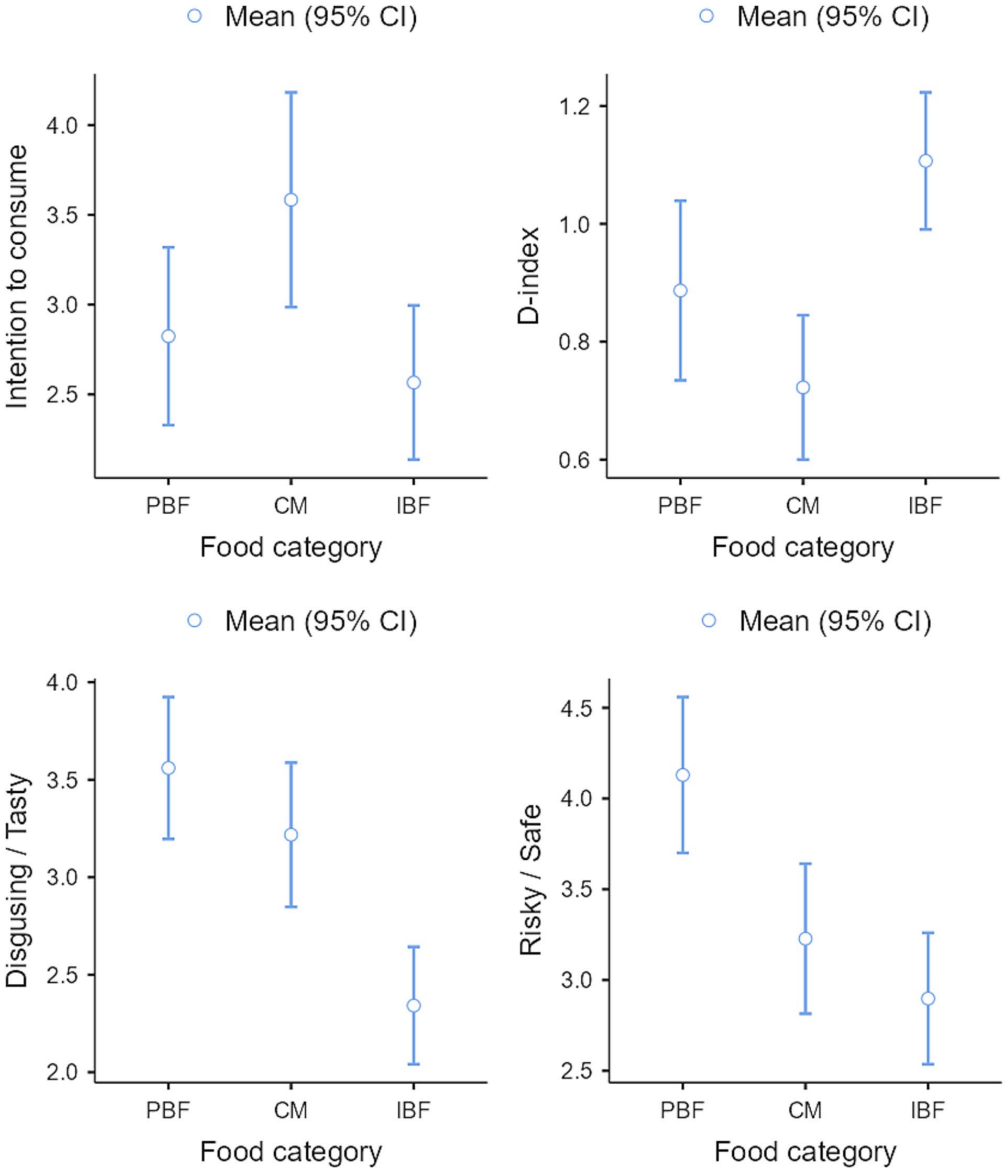
Results of the ANOVAs. The graph in the top left indicates that the intention to consume CM was significantly higher than the intention to consume IBF, while the intention to consume PBF fell in between the two. The graph in the top right shows that the automatic attitudes were more favorable toward CM and PBF than IBF. The bottom left graph reveals that IBF was rated significantly more disgusting than CM and PBF. Finally, the graph in the bottom right illustrates that IBF and CM were rated significantly less safe than PBF.

The last ANOVA focused on the automatic attitude (D-index) as the dependent variable. Assumption checks indicated that the variances across groups were homogeneous [Levene’s test (2,242) = 0.36; *p* > 0.05]. The results revealed a significant effect of food category [Fisher’s *F*(2, 242) = 10.48; *p* < 0.001; *f* = 0.17]. The automatic attitude was more favorable toward CM (Mean = 0.72; SD = 0.61) and PBF (Mean = 0.89; SD = 0.55) than IBF (Mean = 1.11; SD = 0.57). Only the contrast between CM and IBF was significant. Figure 2 illustrates the ANOVA results.

## 4 Discussion

### 4.1 RQ1—predictors of the intention to consume APS

A first important finding concerns the presence of distinct and specific configurations of factors underlying the intention to consume different APS. Considering animal-based APS, the ITC was explained only by explicit factors: disgust for IBF, and both disgust and safety for CM. IBF are typically associated with feelings of disgust because they are not perceived as edible products but rather as pests that live in proximity to dirt and contamination (78). Such representations have also been confirmed in older populations. A qualitative study involving participants over the age of 60 (56) showed that disgust derived from descriptions of insects’ body parts in motion, thus reinforcing the perception of insects as pests rather than edible animals (63). Openness to their consumption in survival scenarios was also mentioned, and this finding was replicated in a more recent survey-based study (35), which confirmed strong reluctance among the older adults.

Regarding perceived risk, although it was relatively high—as discussed in the following section—it does not appear to play a decisive role. A study by La Barbera and colleagues (47) specifically investigating the role of risk perception in the intention to consume found that, although it correlated with behavioral intention, it did not add incremental validity when included in a predictive model. In other words, while risk perception has been identified as a meaningful factor, the authors suggested that in the case of IBF, other attitudes were sufficient for predicting consumers’ intention (i.e., disgust, interest, and attitudes toward entomophagy for feeding other animals) (47).

In the case of cultured meat, technological aspects and the perception of unnaturalness (66, 79, 80) appear to be the primary drivers of both disgust (33) and perceived risk (81, 82). Cultured meat is often viewed as a transgression of traditional methods of meat production (33), which further reinforces negative affective responses and skepticism regarding its safety.

In the case of PBF, the most decisive factor was familiarity. Prior research has already established familiarity as a critical factor, indicating that access to information and previous exposure significantly contribute to more positive attitudes and behavioral intention toward alternative meat products (55, 83). The results of a recent survey (84) highlighted that participants with previous purchase experience were more likely to categorize PBF as a good alternative to conventional meat. This result is particularly relevant in relation to future intervention as it suggests that direct exposure to products may serve as a key driver in fostering behavioral intention.

Besides familiarity, taste-related attitudes proved to explain willingness to introduce PBF in the diet significantly. This finding aligns with previous literature highlighting a significant barrier to transitioning to more vegan consumption, namely expectations that PBF will taste markedly worse than traditional products (85). Interestingly, another recent study on tastiness expectations (86) confirmed this evidence and attributed it mainly to two factors: attachment to the dominant group values of a traditional omnivore diet, and the perception of intergroup threat posed by veganism. These barriers could be very prominent in our specific sample.

Moreover, automatic attitudes emerged as the last explanatory factor of the intention to consume PBF. This result is peculiar since it is present only for this specific food category. Previous research suggests that explicit attitudes are highly shaped by social and cultural norms, while automatic attitudes are more influenced by associative and affective mechanisms (36, 87, 88), differentiating between a top-down vs. a bottom-up process (89). It is therefore plausible that, for PBF, both explicit, top-down factors and automatic, bottom-up factors contribute to explaining intention, whereas for animal-based alternative proteins, explicit determinants play a more prominent role. This finding may appear counterintuitive in light of the previously discussed relevance of disgust and risk perception. However, it can be assumed that, at the automatic level, responses were relatively homogeneous in animal-based APS, since all participants were unfamiliar with the specific product. As a result, explicit evaluations emerged as the most influential predictors. By contrast, in the case of PBF, which were assessed by a more heterogeneous group, the modulation of automatic reactions may also have been reflected in a corresponding modulation of intention.

In all analyses discussed in this section, the role of gender was controlled for, as most previous studies suggest that men exhibit a greater openness to alternative protein sources. This trend appears to be more pronounced for insect-based foods (IBF) (3, 35, 42) than for cultured meat (CM) (3, 30) or plant-based foods (PBF) (3). However, this study did not find a significant relationship between gender and the intention to consume alternative protein sources (APS). This may be explained by the fact that the influence of gender may be overshadowed by other, more relevant variables in the regression models, as noted in previous studies (30).

### 4.2 RQ2—APS comparison of attitudes and intention to consume

From the second set of analyses, it is evident that, although differences emerged across food categories, all three types of alternative protein sources were evaluated rather negatively, both in terms of intention and attitudes. On a scale from 1 to 10 indicating the likelihood of future consumption, none of the products reached a score higher than 4.

A direct comparison between categories confirmed some of the findings already suggested in the previous section. Specifically, insect-based foods received the lowest ratings on the disgusting/tasty scale compared to both plant-based foods and cultured meat, while both animal-based sources (IBF and CM) scored lower than PBF in relation to risk perception. Regarding explicit attitudes, PBF appeared to be the most positively evaluated category among participants. This finding is consistent with the existing literature, which has already positioned PBF as the preferred source of alternative proteins, both among the general population (84, 90) and among older adults (3).

What distinguishes our findings from previous evidence, however, is that this apparently more positive disposition toward PBF does not translate into the intention to consume and is not mirrored by automatic attitudes. On the contrary, automatic attitudes toward PBF explain ITC and reveal an opposite trend, with a gradient from CM, rated as the most likely to be consumed, and IBF, the least likely option. Plant-based foods occupy an intermediate position. As already discussed, prior familiarity and knowledge of the products generally facilitate more favorable attitudes (61, 62). However, in this analysis, only participants who had never tasted PBF were included. It is therefore plausible that, in older adults, and particularly in this subgroup lacking familiarity, skepticism against PBF could be present, since PBF were perceived as less likely to be consumed than what is formally considered a novel food, namely cultured meat.

In addition to the role of familiarity, another important factor to consider is the attachment to a traditional diet. Previous studies have shown that shifting toward a more plant-based diet is often associated with a sense of loss and sacrifice (91, 92) and prevented by the enjoyment of meat and an unwillingness to alter eating habits (93). This feeling may be even more pronounced in older adults, whose protein intake largely derives from animal-based sources (94). A cross-sectional study conducted in five EU countries (95) revealed that consumers aged 65 and older were particularly attached to their consumption of red meat, processed meat, and poultry, and showed little intention to change their diets. Specifically, only between 7 and 17% expressed a willingness to increase their consumption of plant-based substitutes (95, 96). Moreover, the same study, using a choice experiment, demonstrated that older European consumers would rather abstain from eating hamburgers than consume an enriched option with plant-based proteins, while hamburgers enriched with red meat or poultry proteins were preferred over not eating hamburgers at all.

Taken together, these insights suggest that our specific sample, characterized by a lack of familiarity with PBF, may have been more inclined to overlook the concerns associated with cultured meat production techniques, while displaying greater reluctance toward products that imitate meat. In this sense, items such as tofu or seitan may have been perceived in a similar way to novel foods, despite their longer market presence. This result may also be consistent with a recent Italian review on consumer acceptance of novel foods (97), which identified meat eaters as the potential consumers of cultured meat (98, 99).

The discrepancy between explicit and automatic attitudes toward PBF may again be explained by the different nature of these two constructs (36, 87–89). It is therefore possible that participants relied on shared and socially transmitted knowledge regarding the safety and tastiness of PBF, while at a deeper level, substantial resistance remained, which may be justified by the more emotional aspects of eating, as previously discussed. Such resistance ultimately translated into reluctance to consume these products.

Some limitations of this study should be acknowledged. First, participants were asked if they had ever tried the products, but not to self-report their knowledge of each APS, which would have had a mediator role. Second, the nature of the foods examined prevented a cleaner design: for IBF and CM, large samples of participants with direct tasting experience were not available. Future studies should compare these three food categories more systematically. Additionally, further investigation could be conducted for PBF, distinguishing between those who have occasionally tasted it and those who regularly consume it.

## 5 Conclusion

The present study contributes to the growing literature on consumer acceptance of APS by targeting an underrepresented segment of the population: older adults. This population is particularly vulnerable to protein-energy malnutrition, and its openness to dietary innovation remains largely unexplored. By examining both explicit and automatic attitudes, alongside familiarity, we identified distinct configurations of explanatory factors of the intention to consume across three APS. Disgust and perceived risk emerged as the primary drivers of animal-based APS, while familiarity, taste-related attitudes, and automatic responses played a central role in the case of plant-based foods. A particularly innovative contribution of this study lies in the finding that, despite being positively evaluated at the explicit level, PBF elicited negative automatic attitudes and low intention to consume in our sample. This discrepancy suggests that, in older adults, PBF may be implicitly resisted and even perceived as less compatible with their dietary habits than cultured meat, a true novel food. Such evidence challenges the prevailing narrative that positions PBF as the most accepted category of APS, highlighting the need to investigate further the implicit barriers that may hinder their integration for healthy aging.

Taken together, these findings not only broaden the understanding of how psychological determinants shape older adults’ openness to APS but also suggest practical directions to guide targeted strategies aimed at both improving protein intake and supporting the sustainable transition in aging populations. For example, interventions fostering direct exposure and familiarity may be key in overcoming skepticism toward PBF. At the same time, communication around CM and IBF should carefully address concerns linked to disgust and perceived risk.

## Data Availability

The raw data supporting the conclusions of this article will be made available by the authors, without undue reservation.

## Footnotes

^1^ https://www.bilendi.it
